# Supplementary material for: NPAS4 supports cocaine-conditioned cues in rodents by controlling the cell type-specific activation balance in the nucleus accumbens
Source: Nat Commun. 2024 Aug 8;15:5971. doi: 10.1038/s41467-024-50099-1 (PMC11310321; doi:10.1038/s41467-024-50099-1)
Supplement: Supplementary file 1 — Supplementary Information [file 41467_2024_50099_MOESM1_ESM.pdf]

Supplementary Information for

NPAS4 supports cocaine-conditioned cues in rodents by controlling the cell type-specific activation balance in the nucleus accumbens

Brandon W. Hughes[1*], Jessica L. Huebschman[1*], Evgeny Tsvetkov[1], Benjamin M. Siemsen[2], Kirsten K. Snyder[1], Rose Marie Akiki[1,3], Daniel J. Wood[1,3], Rachel D. Penrod[1], Michael D. Scofield[2], Stefano Berto[1], Makoto Taniguchi[1, δ], Christopher W. Cowan[1,δ]

*Authors contributed equally to this work
[1]Department of Neuroscience, Medical University of South Carolina, Charleston, SC
[2]Department of Anesthesiology, Medical University of South Carolina, Charleston, SC
[3]Medical Scientist Training Program, Medical University of South Carolina, Charleston, SC

[δ]Correspondence: cowanc@musc.edu and taniguch@musc.edu

Documents in this PDF:

- **Figure S1 – Extended Data for Figure 1.** Validation of NPAS4-TRAP mice, representation of viral placements for TRAP experiments, TRAP CPP supporting data, quantification of 4OHT-dependent viral labeling of NPAS4-expressing cells, and cocaine conditioning-dependent reactivation of endogenous NPAS4 after cocaine or saline conditioning TRAP.
- **Figure S2 – Extended Data for Figure 2.** Validation of Npas4-TRAP x Ai14 mice, quantification of NPAS4 protein in D1-tdTomato x D2-eGFP mice, single nuclei RNA-seq quality check and cluster comparison to previously published NAc data, and functional enrichment analysis of DEGs between Npas4+ and Npas4- cells.
- **Figure S3 – Extended Data for Figure 3A-D.** Validation of Cre-dependent NPAS4 shRNA, representation of viral placements for shRNA experiments, shRNA CPP supporting data, locomotor sensitization, and replication of a key finding with a second sequence-independent shRNA.
- **Figure S4 – Extended Data for Figure 3E-I.** Acquisition, extinction, and cocaine-primed reinstatement following NPAS4 knockdown in D1- or D2-MSNs, representation of viral placement for rat SA experiments, and acquisition, extinction, and reinstatement of sucrose self-administration following NPAS4 knockdown in D2-MSNs.
- **Figure S5 – Extended Data for Figure 4 and 5.** Quantification of FOS+ cells following NPAS4 knockdown in D2-MSNs, single nuclei RNA-seq quality check, cluster comparison to previously

published NAc data, and quantification of DEGs within cell-type clusters for different group comparisons.

- **Figure S6 – Extended Data for Figure 6.** Single nuclei RNA-sequencing of the mouse NAc after NPAS4 knockdown and cocaine CPP in D1-MSNs and comparison between D1- and D2-based clusters.
- **Figure S7 – Extended Data for Figure 7.** Spine head diameter analysis in D2-MSNs, representative image of PrL→NAcore virus placements, CPP Pre-Test scores, and NPAS4 knockdown in D2-MSNs, but not D1-MSNs, affects spontaneous EPSC frequency.

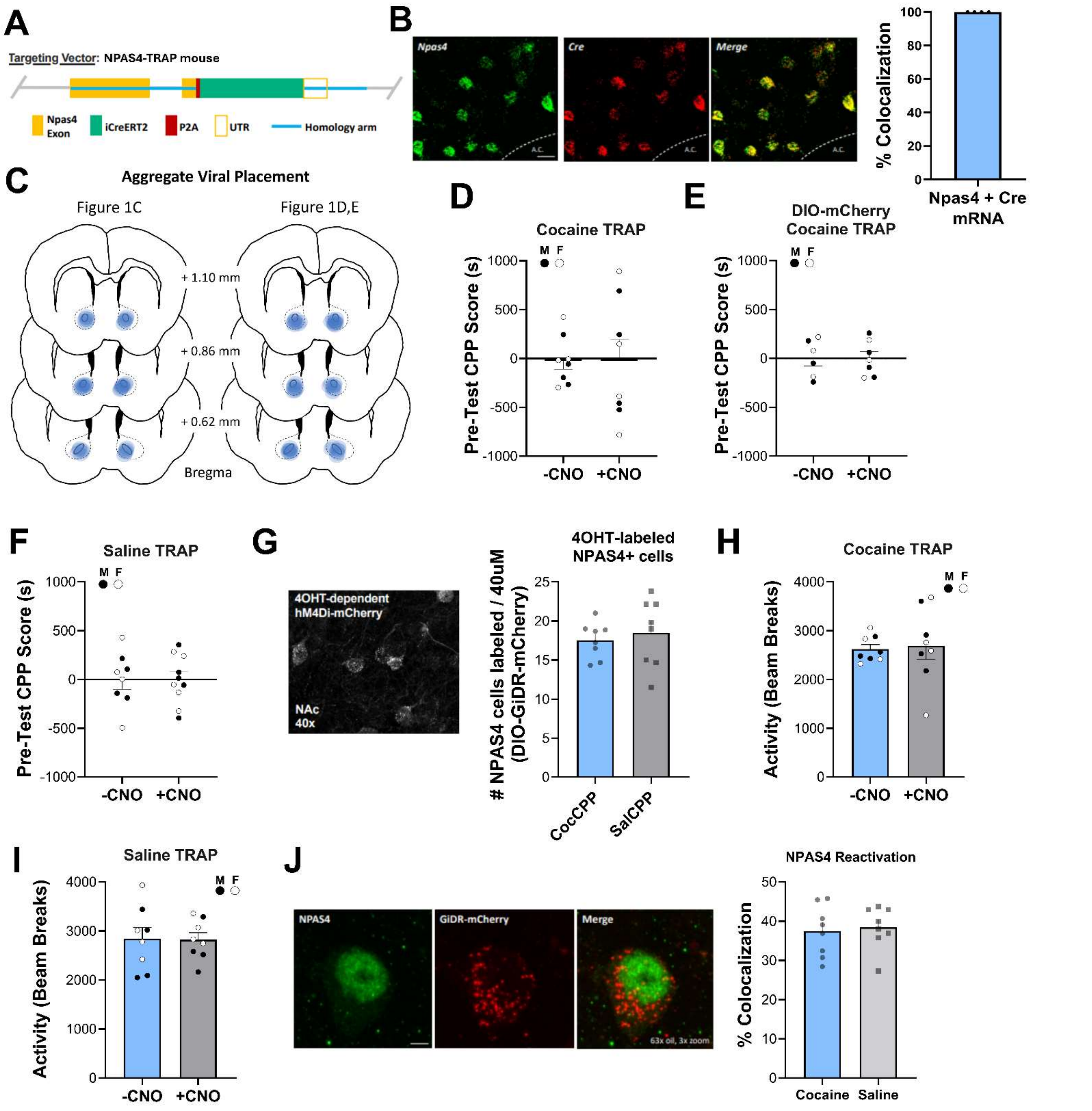

Figure S1 – Extended Data for Figure 1: (**A**)Targeting vector design for CRISPR/Cas9 generation of new NPAS4-TRAP mice. (**B**) Representative image and quantification of *Npas4* mRNA colocalization with Cre following cocaine CPP as validation of NPAS4-TRAP mice. (**C**) Representative NAc-targeted viral expression (A/P: +1.6; M/L: +1.5. D/V: -4.4, from bregma). Pre-test CPP scores for cocaine (**D**), mCherry control (**E**), and saline (**F**) TRAP experiments. (**G**) Representative image *(left)* of 4OHT-dependent mCherry-labeled neurons that expressed NPAS4 during cocaine conditioning and quantification *(right)* of the number of 4OHT mCherry-labeled cells during cocaine and saline conditioning. (**H**) Activity after 4OHT labeling during cocaine CPP and (**I**) saline CPP, both showing no effect of post-test CNO on total locomotion. (**J**) Representative image (*left*) and quantification (*right*) of the colocalization of NPAS4 expression in previously 4OHT mCherry-labelled cells following an additional cocaine or saline conditioning session as a measure of ensemble reactivation. *Data are shown as mean ± SEM. See Source Data File and Detailed Statistical Analysis Table.*

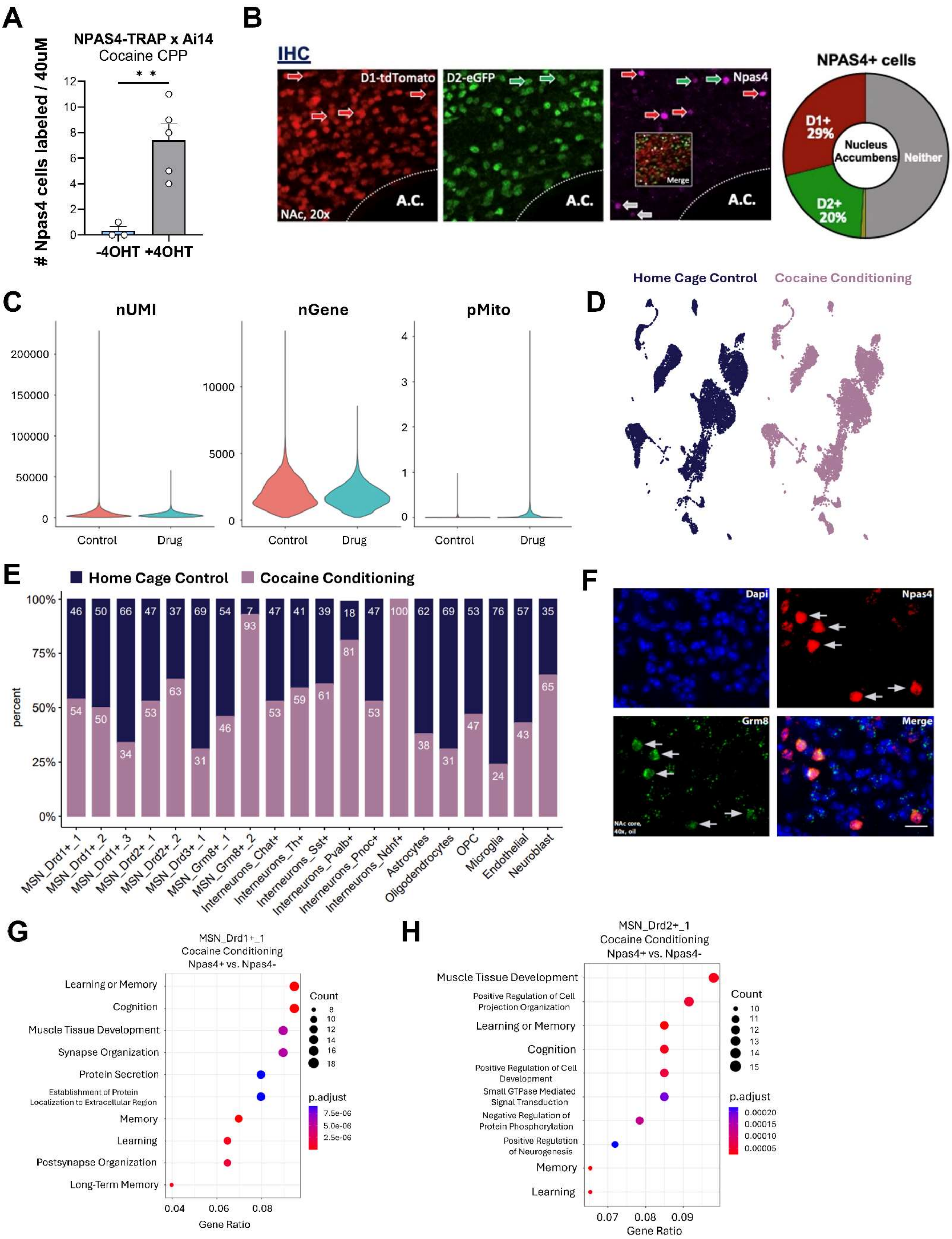
A
NPAS4-TRAP x Ai14
Cocaine CPP
Npas4 cells labeled / 40uM
-4OHT +4OHT
B
IHC
D1-tdTomato
D2-eGFP
Npas4
Merge
NAc, 20x
A.C.
NPAS4+ cells
D1+ 29%
D2+ 20%
Nucleus Accumbens
Neither
C
nUMI
nGene
pMito
Control
Drug
D
Home Cage Control
Cocaine Conditioning
E
Home Cage Control
Cocaine Conditioning
percent
MSN_Drd1+_1
MSN_Drd1+_2
MSN_Drd1+_3
MSN_Drd2+_1
MSN_Drd2+_2
MSN_Drd3+_1
MSN_Grm8+_1
MSN_Grm8+_2
Interneurons_Chat+
Interneurons_Th+
Interneurons_Sst+
Interneurons_Pvalb+
Interneurons_Pnoc+
Interneurons_Ndnf+
Astrocytes
Oligodendrocytes
OPC
Microglia
Endothelial
Neuroblast
F
Dapi
Npas4
Grm8
Merge
NAc core, 40x, oil
G
MSN_Drd1+_1
Cocaine Conditioning
Npas4+ vs. Npas4-
Learning or Memory
Cognition
Muscle Tissue Development
Synapse Organization
Protein Secretion
Establishment of Protein Localization to Extracellular Region
Memory
Learning
Postsynapse Organization
Long-Term Memory
Count
p.adjust
Gene Ratio
H
MSN_Drd2+_1
Cocaine Conditioning
Npas4+ vs. Npas4-
Muscle Tissue Development
Positive Regulation of Cell Projection Organization
Learning or Memory
Cognition
Positive Regulation of Cell Development
Small GTPase Mediated Signal Transduction
Negative Regulation of Protein Phosphorylation
Positive Regulation of Neurogenesis
Memory
Learning
Count
p.adjust
Gene Ratio

Figure S2 – Extended Data for Figure 2: (**A**) Number of labeled cells in the NAc of NPAS4-TRAP x Ai14 mice with or without 4OHT administration immediately after cocaine conditioning. (**B**) Representative images (*left*) and quantification (*right*) showing colocalization of cocaine-induced NPAS4 with D1- or D2-MSNs in a D1-tdTomato x D2-eGFP mouse model. (**C**) Violin plots showing the numbers of UMIs, total genes, and mitochondrial genes in each group. (**D**) UMAPs showing similar cluster distributions between groups and similar numbers of each cell type (**E**) in each group. (**F**) Fluorescent *in situ* hybridization confirming *Npas4* mRNA expression in NAc cells expressing Grm8 mRNA (i.e., Npas4+ Grm8-MSNs). (**G**) Functional enrichment analysis of Npas4+ vs Npas4- Drd1+_1 and (**H**) Drd2+_1 cells 15 min after cocaine conditioning. *Data are shown as mean ± SEM; **p < 0.01. See Source Data File (A,B), Data Availability statement on source data (C-E,G-H), and Detailed Statistical Analysis Table.*

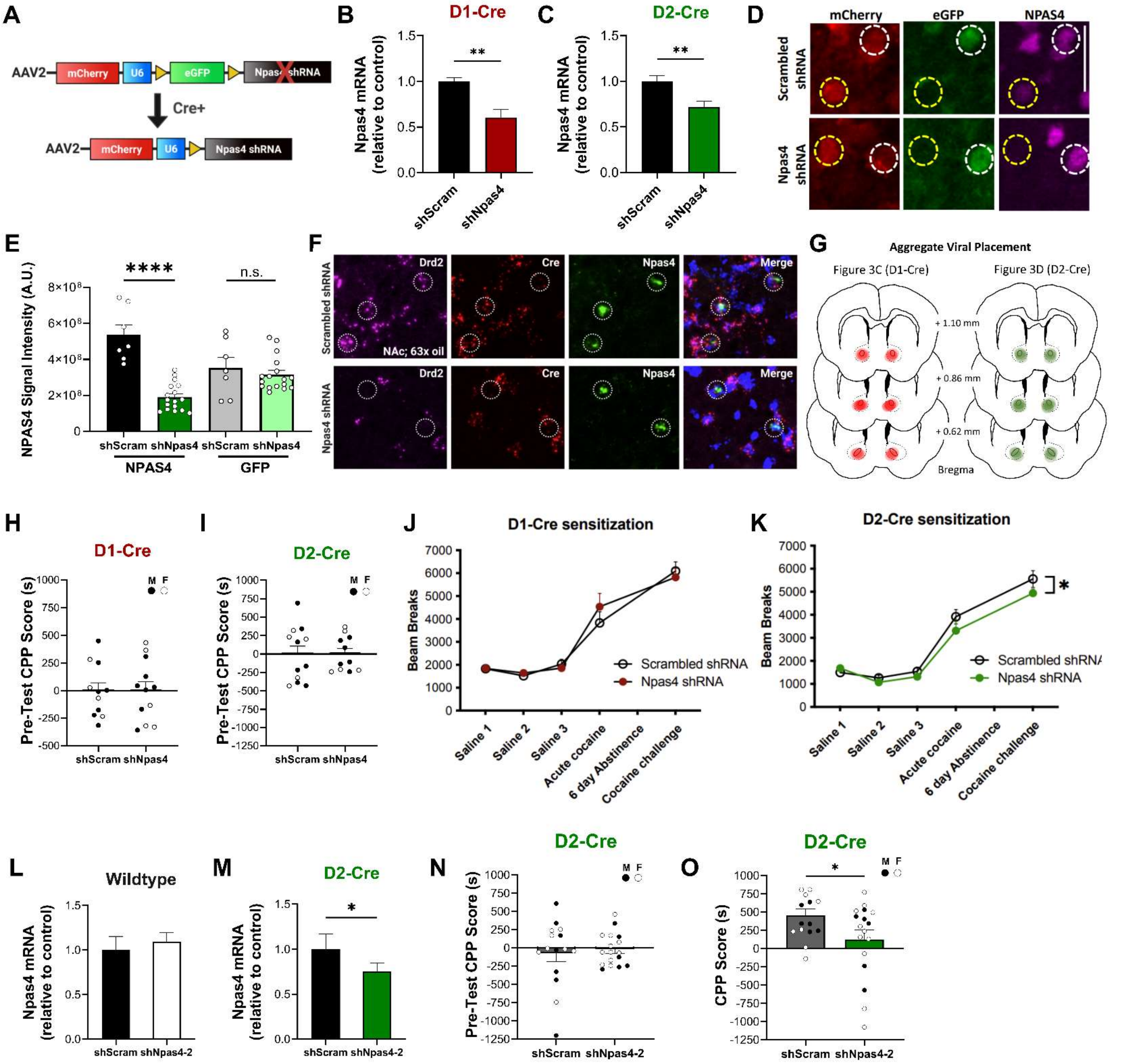

Figure S3 – Extended Data for Figure 3A-D: (**A**) Vector design of Cre-dependent NPAS4 shRNA. (**B-C**) qPCR validation of NPAS4 knockdown in D1-MSNs of D1-Cre mice and D2-MSNs of D2-Cre mice. (**D-E**) Representative image and quantification showing Cre-dependent NPAS4 protein (detected by immunohistochemistry) knockdown with no change in eGFP expression in shScram vs. shNPAS4. (**F**) RNAscope showing Cre-dependent NPAS4 knockdown in D2-Cre mice. (**G**) Representation of viral targeting in D1- (left) and D2-Cre (right) mice. (**H-I**) Pre-test CPP scores for D1- and D2-Cre shNpas4 experiments. (**J-K**) Locomotor sensitization following NPAS4 knockdown in D1-MSNs and D2-MSNs. (**L-M**) qPCR validation of a second Cre-dependent shRNA targeting Npas4, with no change in Npas4 expression in wild-type animals, but a significant reduction in D2-Cre mice. Pre- (**N**) and post-test (**O**) CPP scores for D2-Cre mice with Npas4 knockdown driven by the second shRNA. *Data are presented as mean ± SEM; *p < 0.05, **p < 0.01, ****p < 0.0001, ns = not significant. See Source Data File and Detailed Statistical Analysis Table.*

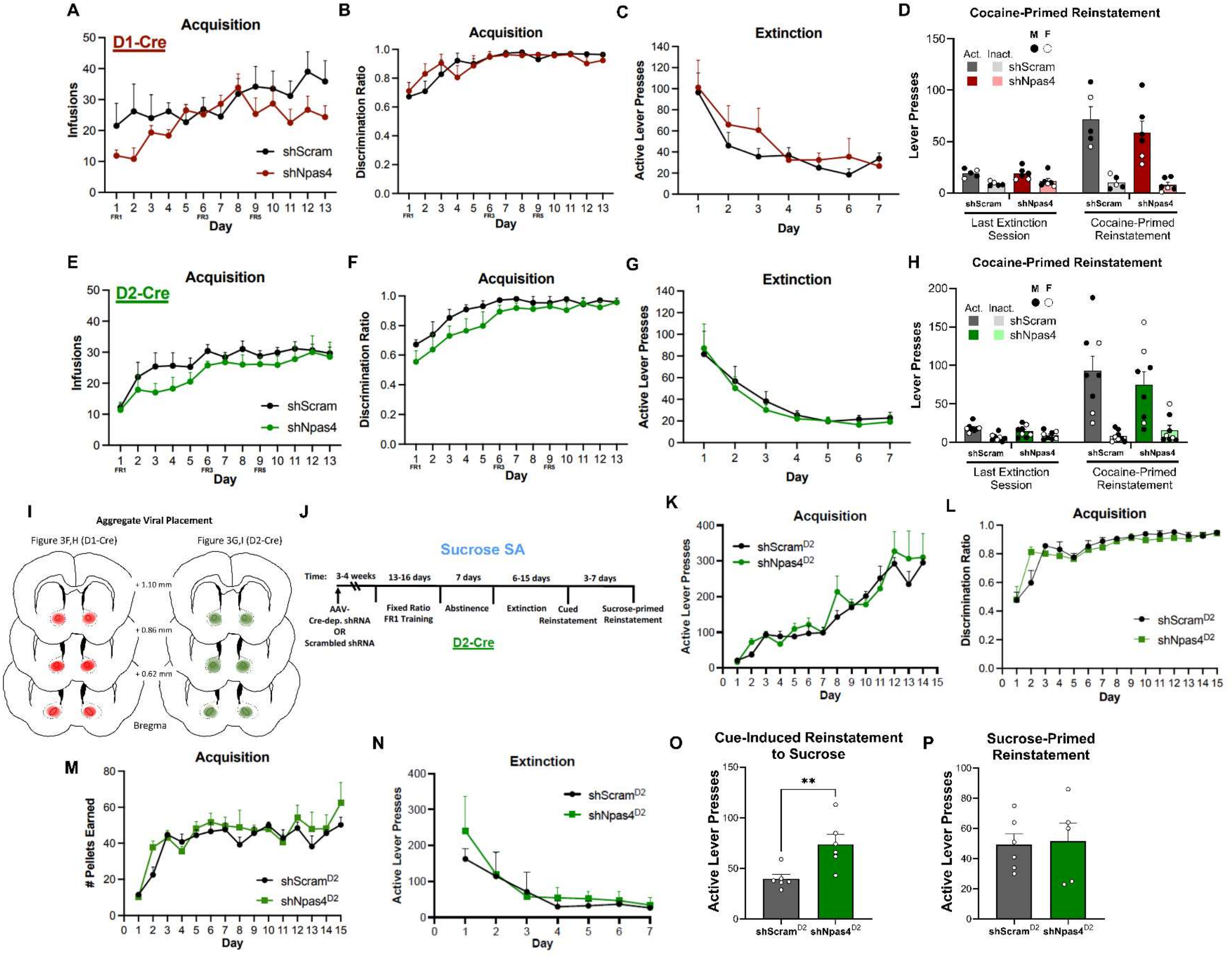

Figure S4 – Extended Data for Figure 3E-I. (**A, E**) The number of infusions received and (**B, F**) the lever discrimination ratio for D1- and D2-Cre rats during cocaine SA acquisition following cell type-specific NPAS4 knockdown compared to controls. (**C, G**) Active lever presses performed by D1-Cre rats during extinction. (**D, H**) Cocaine-primed reinstatement in D1- and D2-Cre rats following cell type-specific knockdown. (**I**) Representative viral placements for rat SA studies. (**J**) Timeline of sucrose SA after NPAS4 knockdown in D2-Cre rats. (**K-M**) Active lever presses, discrimination ratio, and the number of pellets earned during acquisition. (**N**) Active lever presses made during extinction. (**O**) Cue-induced and (**P**) sucrose-primed reinstatement to sucrose seeking in D2-Cre rats following cell type-specific knockdown of NPAS4. *Data are shown as mean ± SEM; **p < 0.01. See Source Data File and Detailed Statistical Analysis Table.*

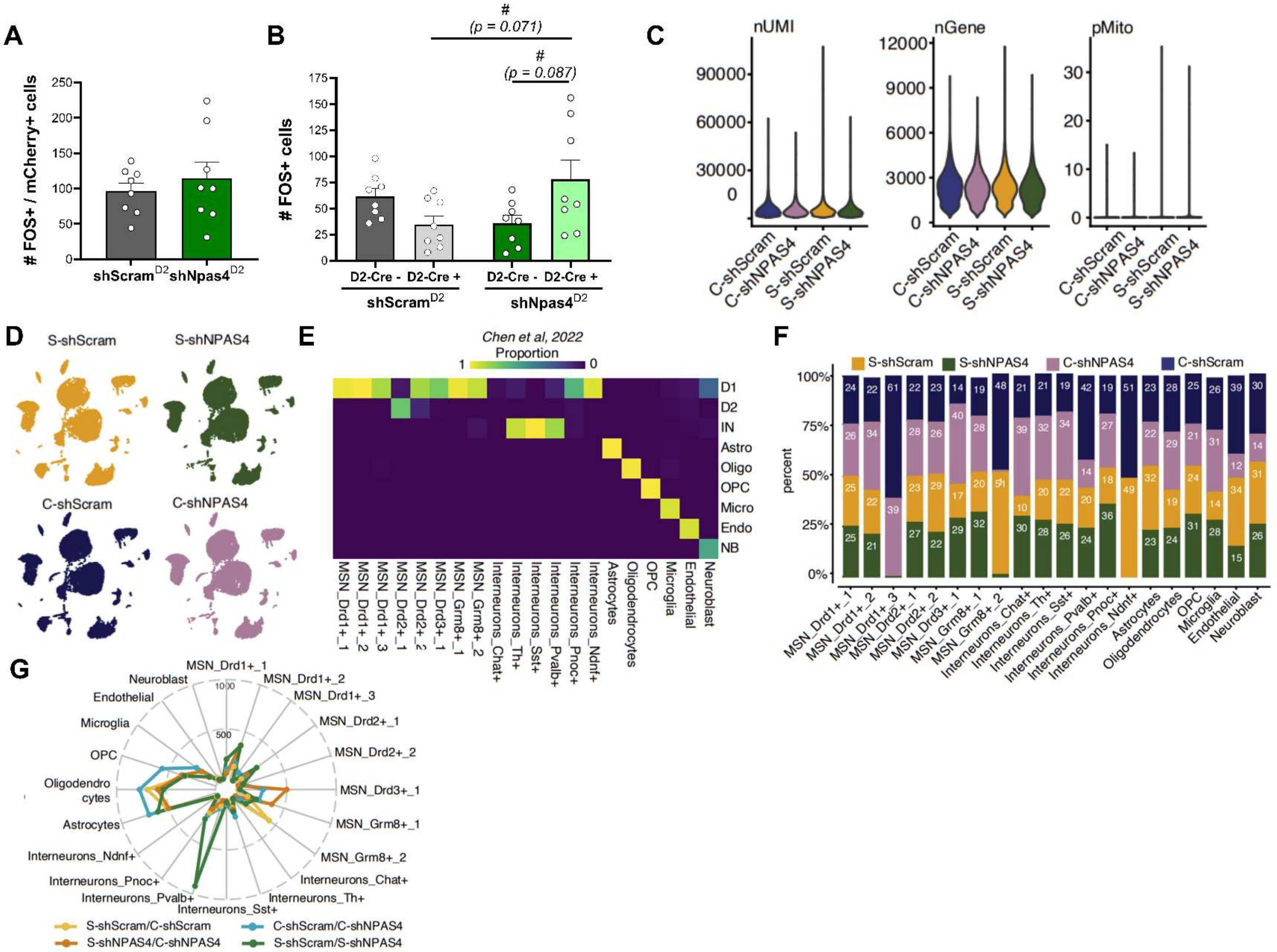

Figure S5 – Extended Data for Figure 4 and 5. (**A**) Total number of FOS and mCherry positive cells, showing no effect of virus on overall number of FOS positive virally infected cells. (**B**) Number of FOS positive NAc D2-Cre negative and D2-Cre positive cells with knockdown of NPAS4 in D2-MSNs. (**C**) Violin plots showing the numbers of UMIs, total genes, and mitochondrial genes in each group. (**D**) UMAPs showing similar cluster distributions between groups and (**E**) similar numbers of each cell type in each group. (**F**) Overlap of defined clusters from this study compared to Chen et al., 2021. (**G**) Radar plot showing the number of differentially expressed genes in each cluster for each group comparison. *Data are presented as mean ± SEM; *p < 0.05, **p < 0.01, ****p < 0.0001, ns = not significant. See Source Data File (A-B), Data Availability statement on source data (C-G), and Detailed Statistical Analysis Table.*

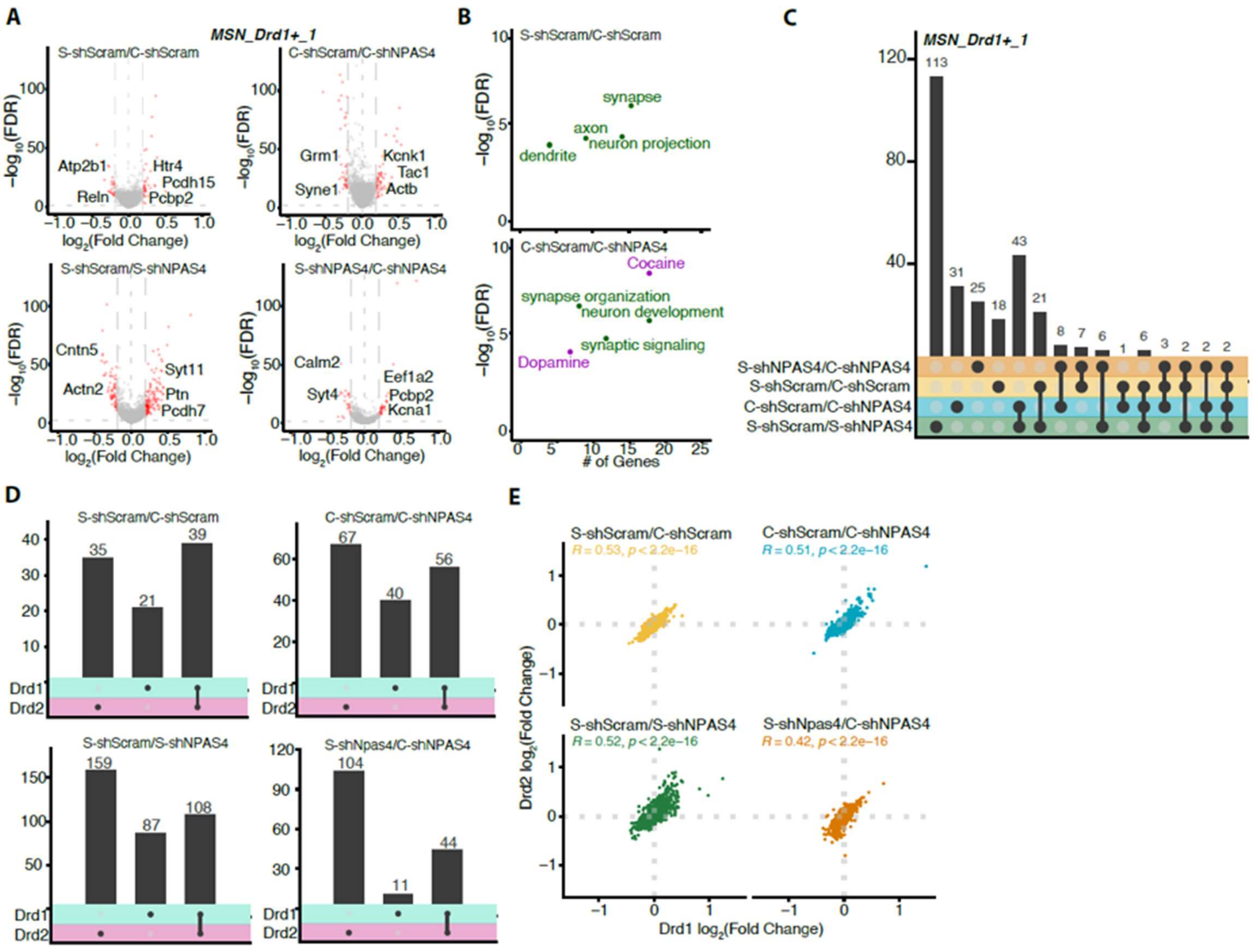

Figure S6 – Extended Data for Figure 6. (**A**) Volcano plots depicting D1-MSN-specific DEGs in cocaine CPP vs saline CPP mice +/- NPAS4. (**B**) Gene ontology analysis of DEGs in D2-MSNs comparing (*top*) "Saline CPP" vs "Cocaine CPP" and (*bottom*) "Cocaine CPP" vs "Cocaine CPP + shNPAS4." The X-axis depicts the number of genes in each category, whereas the Y-axis corresponds to -log10(FDR) based on Fisher's exact test. (**C**) Upset plot showing the number of overlapping DEGs between groups in D1-MSNs. (**D**) Upset plots showing DEGs specific to D1-MSNs, D2-MSNs, or both cell types. (**E**) Correlation plots depicting the concordance between fold changes of DEGs in D1- and D2-MSNs and significant correlations in all four groups. The correlation values and the relative p-values are colored by group (Spearman rank correlation). *See Data Availability for source data.*

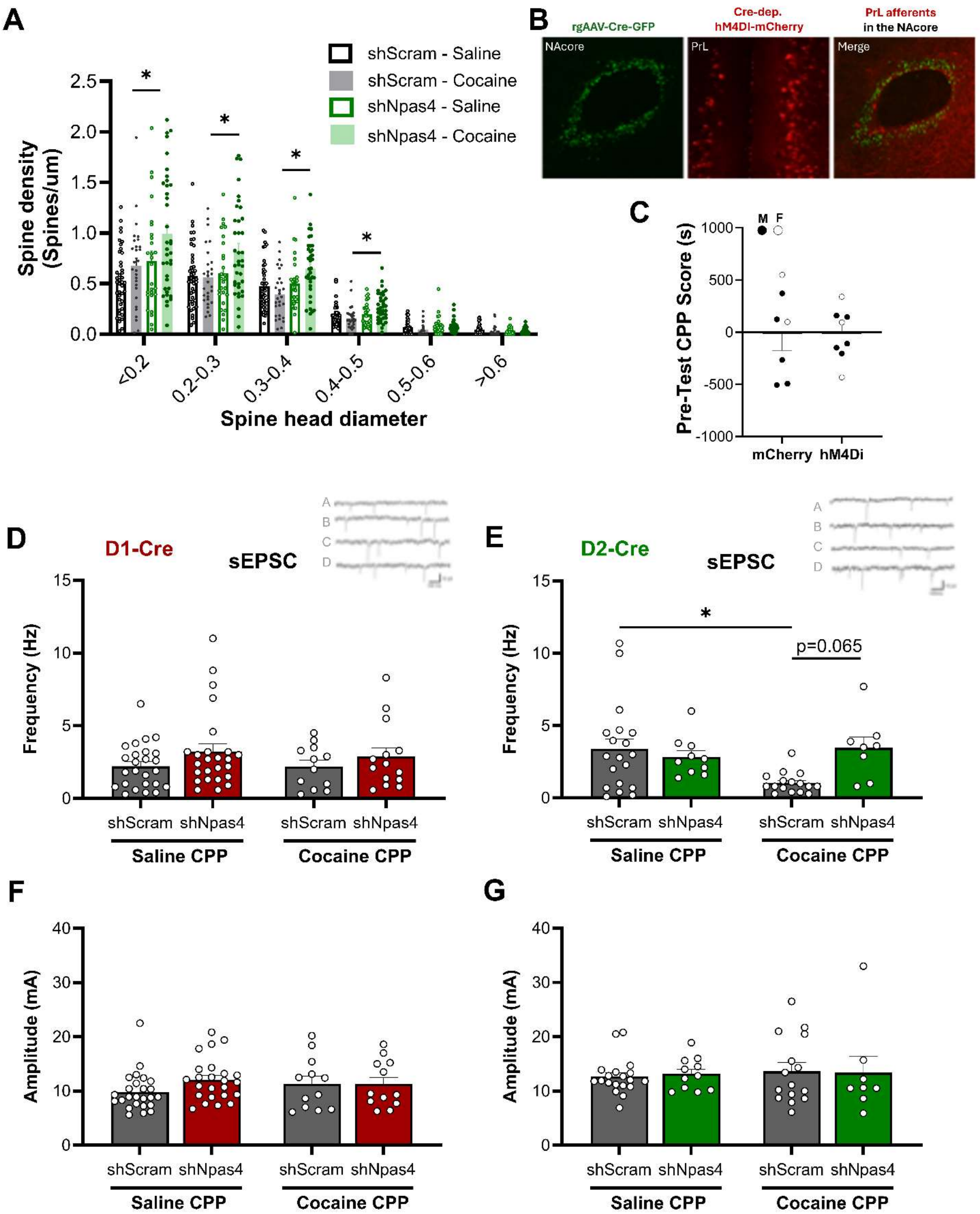

Figure S7 – Extended Data for Figure 7. (**A**) Quantification of spine density in D2-MSNs by spine head diameter. (**B**) Representative image showing AAV-retrograde Cre in the nucleus accumbens core (*green*) and AAV-Cre-dependent hm4Di-mCherry in the prelimbic cortex (*red*). (**C**) Pre-test CPP scores for experiment in Figure 7E. (**D-E**) Spontaneous EPSC frequencies in D1-MSNs and D2-MSNs. (**F-G**) sEPSC amplitudes after NPAS4 knockdown in D1-MSNs (*left*) and D2-MSNs (*right*) following cocaine conditioning. Insets on **D** and **E** show representative traces of spontaneous transmission in the following groups: (A) S-shScram, (B) S-shNPAS4, (C) C-shScram, and (D) C-shNPAS4. *Data are shown as mean ± SEM; *p < 0.05. See Source Data File and Detailed Statistical Analysis Table.*
