# Supplementary material for: NPAS4 supports cocaine-conditioned cues in rodents by controlling the cell type-specific activation balance in the nucleus accumbens
Source: Nat Commun. 2024 Aug 8;15:5971. doi: 10.1038/s41467-024-50099-1 (PMC11310321; doi:10.1038/s41467-024-50099-1)
Supplement: Supplementary file 3 — Description of Additional Supplementary Files [file 41467_2024_50099_MOESM3_ESM.pdf]

Description of Additional Supplementary Files

NPAS4 supports cocaine-conditioned cues in rodents by controlling the cell type-specific activation balance in the nucleus accumbens

Brandon W. Hughes[1*], Jessica L. Huebschman[1*], Evgeny Tsvetkov[1], Benjamin M. Siemsen[2], Kirsten K. Snyder[1], Rose Marie Akiki[1,3], Daniel J. Wood[1,3], Rachel D. Penrod[1], Michael D. Scofield[2], Stefano Berto[1], Makoto Taniguchi[1, δ], Christopher W. Cowan[1,δ]

*Authors contributed equally to this work
[1]Department of Neuroscience, Medical University of South Carolina, Charleston, SC
[2]Department of Anesthesiology, Medical University of South Carolina, Charleston, SC
[3]Medical Scientist Training Program, Medical University of South Carolina, Charleston, SC

[δ]Correspondence: cowanc@musc.edu and taniguch@musc.edu

Legends for Supplementary Data Files (external attachments):

- **Supplementary Data 1:** Marker gene statistics for all clusters in the snRNA-seq datasets shown in Fig. 2.
- **Supplementary Data 2:** Differential expression statistics related to Figure S2.
- **Supplementary Data 3:** Marker gene statistics for all clusters in the snRNA-seq datasets are shown in Figure 5.
- **Supplementary Data 4:** Differential expression statistics related to Figures 5 and 6.
- **Supplementary Data 5:** Details for All Statistical Analysis
