# Supplementary material for: NPAS4 supports cocaine-conditioned cues in rodents by controlling the cell type-specific activation balance in the nucleus accumbens
Source: Nat Commun. 2024 Aug 8;15:5971. doi: 10.1038/s41467-024-50099-1 (PMC11310321; doi:10.1038/s41467-024-50099-1)
Supplement: Supplementary file 9 — Reporting Summary [file 41467_2024_50099_MOESM9_ESM.pdf]

nature portfolio

Corresponding author(s): Christopher W. Cowan, Makoto Taniguchi

Last updated by author(s): Jun 2, 2024

Reporting Summary

Nature Portfolio wishes to improve the reproducibility of the work that we publish. This form provides structure for consistency and transparency in reporting. For further information on Nature Portfolio policies, see our Editorial Policies and the Editorial Policy Checklist.

Statistics

For all statistical analyses, confirm that the following items are present in the figure legend, table legend, main text, or Methods section.

n/a	Confirmed	
☐	☒	The exact sample size (*n*) for each experimental group/condition, given as a discrete number and unit of measurement
☐	☒	A statement on whether measurements were taken from distinct samples or whether the same sample was measured repeatedly
☐	☒	The statistical test(s) used AND whether they are one- or two-sided<br>*Only common tests should be described solely by name; describe more complex techniques in the Methods section.*
☒	☐	A description of all covariates tested
☐	☒	A description of any assumptions or corrections, such as tests of normality and adjustment for multiple comparisons
☐	☒	A full description of the statistical parameters including central tendency (e.g. means) or other basic estimates (e.g. regression coefficient) AND variation (e.g. standard deviation) or associated estimates of uncertainty (e.g. confidence intervals)
☐	☒	For null hypothesis testing, the test statistic (e.g. *F*, *t*, *r*) with confidence intervals, effect sizes, degrees of freedom and *P* value noted<br>*Give P values as exact values whenever suitable.*
☒	☐	For Bayesian analysis, information on the choice of priors and Markov chain Monte Carlo settings
☐	☒	For hierarchical and complex designs, identification of the appropriate level for tests and full reporting of outcomes
☐	☒	Estimates of effect sizes (e.g. Cohen's *d*, Pearson's *r*), indicating how they were calculated

Our web collection on statistics for biologists contains articles on many of the points above.

Software and code

Policy information about availability of computer code

Data collection	R v4.1.2, Prism v9.3.0 (GraphPad)
Data analysis	Cellranger (v6.1.2) for single nuclei RNA-seq analysis. Cellbender (v0.232) for RNA ambient removal. R package Suerat (v4.1.0) for downstream clustering analysis. scDBIFinder (v1.8.0) to identify doublets. scPred (v1.9.2) for predictive cell annotation using a reference based data. LIBRA (v1.0.0) for the differential expression analysis based on MAST. The analysis code described in this study is available at https://github.com/BioinformaticsMUSC/HughesEtAl_NPAS4Cocaine/<br>https://zenodo.org/doi/10.5281/zenodo.11265104

For manuscripts utilizing custom algorithms or software that are central to the research but not yet described in published literature, software must be made available to editors and reviewers. We strongly encourage code deposition in a community repository (e.g. GitHub). See the Nature Portfolio guidelines for submitting code & software for further information.

Data

Policy information about availability of data

All manuscripts must include a data availability statement. This statement should provide the following information, where applicable:

- Accession codes, unique identifiers, or web links for publicly available datasets
- A description of any restrictions on data availability
- For clinical datasets or third party data, please ensure that the statement adheres to our policy

All processed sequencing data, UMAP coordinates, and annotations have been made freely available to download at the BioCM portal:
https://github.com/BioinformaticsMUSC/HughesEtAl_NPAS4Cocaine.
Interactive sequencing data is available through ShinyCell at:
https://bioinformatics-musc.shinyapps.io/Jessica_NAc_Cocaine_Npas4/
https://bioinformatics-musc.shinyapps.io/Hughes_NAc_Cocaine_NPAS4/
Raw and processed sequencing data to support the findings of this study have been deposited in GEO under accession number: GSE210850.
Other source data are provided with this paper in the Source Data file.

Research involving human participants, their data, or biological material

Policy information about studies with human participants or human data. See also policy information about sex, gender (identity/presentation), and sexual orientation and race, ethnicity and racism.

Reporting on sex and gender	This research did not involve human participants.
Reporting on race, ethnicity, or other socially relevant groupings	This research did not involve human participants.
Population characteristics	This research did not involve human participants.
Recruitment	This research did not involve human participants.
Ethics oversight	This research did not involve human participants.

Note that full information on the approval of the study protocol must also be provided in the manuscript.

Field-specific reporting

Please select the one below that is the best fit for your research. If you are not sure, read the appropriate sections before making your selection.

☒ Life sciences ☐ Behavioural & social sciences ☐ Ecological, evolutionary & environmental sciences

For a reference copy of the document with all sections, see nature.com/documents/nr-reporting-summary-flat.pdf

Life sciences study design

All studies must disclose on these points even when the disclosure is negative.

Sample size	Sample sizes for all experiments were estimated based on prior power analysis of similar study designs.
Data exclusions	No data were excluded from the analyses.
Replication	Findings were not replicated due to the uniqueness of the data analyzed.
Randomization	Samples were randomized at each stage of data collection. All covariates including batch effects due to randomization were taken into account.
Blinding	Data collection and analyses were performed by experimenters blinded to experiment conditions. In addition, separate individuals carried out wet-bench and dry-bench analyses.

Reporting for specific materials, systems and methods

We require information from authors about some types of materials, experimental systems and methods used in many studies. Here, indicate whether each material, system or method listed is relevant to your study. If you are not sure if a list item applies to your research, read the appropriate section before selecting a response.

Materials & experimental systems

n/a	Involved in the study	
☐	☒	Antibodies
☒	☐	Eukaryotic cell lines
☒	☐	Palaeontology and archaeology
☐	☒	Animals and other organisms
☒	☐	Clinical data
☒	☐	Dual use research of concern
☒	☐	Plants

Methods

n/a	Involved in the study	
☒	☐	ChIP-seq
☒	☐	Flow cytometry
☒	☐	MRI-based neuroimaging

Antibodies

Antibodies used

rabbit-NPAS4 (kindly gifted from Dr. Michael Greenberg's Lab; 1:1000), rabbit-cFOS (#226009, Synaptic Systems, 1:1000), rabbit-FLAG (#F2555, Sigma Aldrich, 1:1000), species-specific secondary antibodies produced in donkey and conjugated to Alexa Fluor 488, Alexa Fluor 555, or Alexa Fluor 647 (Thermo Fisher Scientific, 1:500). RNAscope probes (ACD Bio) included MmNpas4-C1 (#423431), iCre-C2 (#423321-C2), Mm-Drd1a-C2 (#406491-C2), Mm-Drd1a-C3 (#406491-C3), Mm-Drd2-C3 (#406501-C3), and tdTomato-C1 (#317041)

Validation

NPAS4: Lin et al., 2008; Taniguchi et al., 2017
cFOS: Li et al., 2022
FLAG: Siemsen et al., 2022; He et al., 2015; Qungang et al., 2012; Banerjee et al., 2017; Hamajima et al., 2013; Wang et al., 2017

Animals and other research organisms

Policy information about studies involving animals; ARRIVE guidelines recommended for reporting animal research, and Sex and Gender in Research

Laboratory animals

All experiments were conducted in male and female animals 8-16 weeks of age during their dark phase. All animals were fed ad libitum, unless specified otherwise, and maintained at ~22 degrees Celsius in a humidity controlled environment on a reverse 12-h light/dark cycle. All mice were bred on the C57BL/6J background. Some experiments utilized transgenic mice, including NPAS4-TRAP, Drd1a-Cre (line FK150), Drd2-Cre (line ER44), D1-tTomato, and D2-eGFP, which were obtained from P. Kalivas and NINDS/GENSAT. D1- and D2-Cre rats were obtained from NIDA, and then backcrossed to Charles River Long-Evans rats.

Wild animals

No wild animals were used in this study.

Reporting on sex

All experiments include approximately equal numbers of male and female animals. We observed no effect of sex in these experiments and data is displayed disaggregated by sex where possible.

Field-collected samples

The study did not involve field-collected samples.

Ethics oversight

The Institutional Animal Care and Use Committee (IACUC) at Medical University of South Carolina
The NIDA-IRP Institutional Animal Care and Use Committee of the US National Institutes of Health (NIH)

Note that full information on the approval of the study protocol must also be provided in the manuscript.

Plants

Seed stocks

This research did not involve plants.

Novel plant genotypes

This research did not involve plants.

Authentication

This research did not involve plants.
